# Housing and Child Welfare

**Published:** 1921-03-26

**Authors:** 


### THE PUBLIC WELL-BEING.

### Housing and Child Welfare.

### THE PROBLEM OF THE TENEMENT DWELLING.

In her thoughtful and in many respects valuable study * of the social aspects of the subject of child welfare, Miss Nora Milnes has a chapter on houses to which we should like to call special attention. We cannot agree with the writer's preliminary observation that the evil effects of bad housing conditions are now so clearly recognised that the problem seems not to require quite the same amount of insistence that others demand; but to almost everything that follows we can say a sincere "Amen." It may be generally agreed, in vague acquiescence, that better homes for the people are essential if any efforts at social betterment are to obtain; but it is more than doubtful even now how far the general public is alive to the conditions under which many thousands of families are forced to live. The real problem that faces the woman of the so-called slums is that such hopelessness exists all around her that she quickly "becomes a part of her environment and soon loses the power to strive after anything better."

The only home that she can afford is one in which cleanliness is well-nigh impossible, one that puts to ridicule the frequently heard remark that it costs nothing to be clean. In fact, such a statement can but be made in entire ignorance of the circumstances, and equally in ignorance of the meaning of the word "cost." For cost does not merely imply a financial outlay; it comprises all the pain and toil that have to be undergone in obtaining some desired end. Compelled as many a woman is to live on the top floor of a high tenement dwelling, in which water is not laid on upstairs, and of which, therefore, every drop that is required has to be carried some distance; a tenement in which a supply of hot water emerging from a tap is almost in the world of fairy stories, so that all that is needed has to be heated on a fire that is wanted for other purposes; a tenement consisting of one room, so that there is no place into which the few household possessions can be put while an attempt is made to have as good a scrub as a very inferior brush will permit; how after this can it be contended that, in any reasonable meaning of the word "cost," it costs nothing to be clean?

### The "Marvel" of Survival.

These are incontestable facts, well and simply put, which have to be faced in the effort to save infant life and to produce a healthy race. The author's comparisons are equally to the point when she pauses to consider the ordinary rules of life that are followed by the family that lives not in luxury but in ordinary comfort, and sets down the difficulties that confront the underhoused family.

If the child of the better-provided classes falls ill it is separated from the rest of the family. If the mother is ill her children are kept from her so that she may have the quiet essential if she is to recover quickly. A room is set apart in the house for the children to have as their nursery, and a bright, airy room is invariably chosen. The child is bathed every day. His nursery is cleaned every day while he is elsewhere, probably taking his regular outings that last the greater part of the morning. The purest milk obtainable is kept in as clean a place as possible, and boiled in a special saucepan that is not used for other purposes. These are merely examples of the simplest rules that we should describe as essential for healthy existence. Let us, then, ask ourselves how many of those living in an overcrowded area can follow any one of these rules. If we know anything of their lives we know also that most of these simple needs cannot be supplied. In consequence the wonder is not that children are so weakly, the marvel is that under such environmental conditions so many manage to thrive.

The author applies a number of tests to prove that bad housing conditions are one of the main causes of a high infant-mortality rate, as well as a high rate of physical defect among the children who survive. Dr. Lawson Dick made a careful examination from another standpoint of children attending schools in the East End of London, and the results were convincing. He chose Jewish children for his inquiry, because the Jewish mothers nurse their children more often than do the Gentiles. Of the 1,000 children examined, 81.4 per cent. had been breast-fed for periods varying from twelve months to two years, and yet 80 per cent. of these children were rickety. Very few—only about one-fifth—showed definite signs of bad nutrition, so that rickets could not be attributed to artificial feeding or to insufficient food. Dr. Dick concludes that bad housing conditions accounted for the high percentage of children who suffered from this complaint, which in turn produced many other physical defects. This conclusion he supports by reference to the children of Italy, South Africa, and Australia. The children of these countries are often badly fed, but few of them suffer from rickets. In these countries an outdoor life is led, and there is an abundance of sunshine, whereas the children whom he examined are exposed to different conditions. "The great majority . . . lived in two- or three-roomed houses, frequently in one room, and in one of these rooms the cooking and ordinary household work was carried on. Such conditions will inevitably produce rickets, whether the food be good or bad."

### Into the Future.

The author closely analyses the economic factors in the present deplorable conditions of the housing industry in this country; but, like others who have studied the problem as it has developed since the war, she comes to no very hopeful conclusion, because, owing to the heavy cost of labour and material, and the decreasing output of labour, she sees no practical possibility of the wholesale building reform that is absolutely necessary to the social progress of the country. There are signs, how

* *Child Welfare.* By Nora Milnes, Director of the Edinburgh School of Social Study. 6s. J. M. Dent & Sons.

THE PUBLIC WELL-BEING—*(continued)*.

ever—less plainly visible, perhaps, when she was engaged upon writing this chapter of her book—that in the coming months building activity will be resumed on a very much larger scale than in the past two years. It need not be pretended that this will in any way penetrate to the roots of the question, which involves a complete reconstruction of our ideas about industrial existence, and will necessitate in the enlightened future demolition schemes as vast as the housing projects of a community that desires and intends to be healthy. Such conceptions are impracticable in this debt-ridden age, but it is probable that ultimately the development of electricity will go far towards solving the difficulty. "As 'works' are enabled to move away from an industrial centre, new areas will develop, areas in which the pressure of population will be less and in which houses can be built that can be let at rents that the workers can afford to pay."

In her general conclusions about the welfare of the child, Miss Milnes gives what appears to be a qualified approval to the policy usually described by the pure voluntaryists as "State interference." But she frankly admits, as a reply to the "liberty of the subject" argument, in the case of the child wage-earner, that there is here no true freedom, as the child is necessarily at a disadvantage in making a contract, thus paraphrasing the saying of John Stuart Mill that "freedom of contract, in the case of children, is but another word for freedom of coercion." But it is a remark, let us remember, in advocating the cause of child welfare, which bears a very wide application.

